# Silk-CNT Mediated Fibroblast Stimulation toward Chronic Wound Repair

**DOI:** 10.21926/rpm.1904007

**Published:** 2019-12-18

**Authors:** Naiwei Chi, Shuyao Zheng, Elwin Clutter, Rong Wang

**Affiliations:** Department of Chemistry, Illinois Institute of Technology, Chicago, Illinois 60616, USA

**Keywords:** Fibroblast, silk, carbon nanotube, electrical stimulation, collagen I, collagen III, chronic wound

## Abstract

**Background:**

Diabetic patients suffer from chronic wounds partly due to altered function of fibroblasts. Fibroblasts of diabetic patients synthesize collagen I (COLI) at a much higher level than collagen III (COLIII), resulting in delayed tissue granulation and, consequently, a delay in the overall wound healing process.

**Methods:**

We aimed to revive the matrix protein productivity of diabetic fibroblasts by employing aligned, electrically conductive and biocompatible spider silk-CNT fibers as a cell culture matrix to mediate the electrical stimulation of fibroblasts to induce cell polarization and activation.

**Results:**

A 5.2 and 42.7 fold increase in COLI and COLIII production was induced in diabetic fibroblasts. The stimulated cells synthesized a substantially high level of COLIII to reduce the abnormally high COLI/COLIII ratio, and the matrix metalloproteinases expression was markedly suppressed. The protein expression profile was consistent with favorable wound healing. The modulation effect was also demonstrated in normal fibroblasts of healthy individuals, suggesting that the developed method can be utilized generally for connective tissue repair. Silkworm silk-CNT fibers corroborated similar effects on restoring the function of diabetic fibroblasts.

**Conclusions:**

The approach of using an engineered biopolymer matrix to remedy dysfunctional fibroblasts of patients offers the opportunity of developing personalized cell therapy for noninvasive treatments and inspires the design of multi-functional biometrics for effective tissue regeneration.

## 1. Introduction

Diabetes is a debilitating condition that affects 9.4% of the U.S. population [1]. Twenty percent of patients with diabetes suffer chronic wounds that are painful and lead to a significant number of lower-limb amputations each year [2]. Thus, it is crucial to develop safe, simple and economic treatments methods to avoid amputations that severely impede the quality of life and escalate the cost associated with patient care.

Wound healing is a complex process, initiated by hemostasis followed by inflammation, tissue growth, and tissue remodeling [3]. In diabetic patients, hyperglycemia may lead to prolonged inflammation processes. The elevated blood sugar level invokes a higher level of matrix metalloproteinases (MMPs) in the tissue [4]. MMPs are essential for clearing the wound bed and rapid turnover of growth factors, receptors, and extracellular matrix (ECM) proteins [5–7]; however, elevated MMP level in diabetic patients accelerates the degradation of newly synthesized matrix proteins and disrupts their proper deposition and maturation [8]. MMPs are synthesized by fibroblasts, which are also the primary source of ECM proteins that are essential to the wound healing process [9]. Collagen is the most abundant protein in the ECM. Collagen I (COLI) and collagen III (COLIII) are the predominant types of collagen in connective tissues. Typically, fibrillar COLI offers high tensile strength to tissues, whereas COLIII is found along with COLI in tissues that require increased flexibility and distension [10]. In normal skin tissues, the COLI/COLIII ratio is nearly 2.3–2.5 [11]. During tissue growth in wound healing, COLIII synthesis is enhanced by fibroblasts to form granulation tissues to cover up the cleared wound area [12]. However, the COLI/COLIII ratio is much higher in the tissues of diabetic patients [13]. The demand for synthesis of a significantly higher amount of COLIII is high to rectify the stiff tissue to form granulation tissues for wound repair. Nevertheless, the elevated level of MMPs in diabetic patients causes rapid degradation of newly synthesized proteins, leading to the formation of chronic wounds [14, 15].

Current methods of chronic wound care focus mainly on reducing pain, itching, and minimizing infection and bleeding from the wound, and these methods are often not expected to heal the wound [16]. To overcome the deficiency of growth factors and cytokines and to create an environment similar to that of a normal wound healing, engineered skin substitutes have been developed. However, their usage is undermined because of concerns over infection, antigenicity, and hypoglycemia. Adult stem cells, especially mesenchymal stem cells, have been shown to play an important role in wound healing [17, 18]. However, isolation, expansion and differentiation of these cells involve complicated controls and are laborious, expensive and subject to strict and increasing regulatory demands. Fibroblasts are abundant, easy to harvest, culture and expand *in vitro*. While the use of autologous fibroblasts is simple and economic, a major obstacle in their application is the molecular and cellular abnormality of diabetic fibroblasts. Therefore, it is imperative that the fibroblast functions of the diabetic patient are renewed before they are used for treatment.

A transepithelial potential difference (TEPD), varying from 10 to 60 mV, is present across the epithelium due to an uneven distribution of sodium (Na^+^) ions [19].When the skin is damaged, this Na^+^ gradient is broken, leading to the increased electric field in the region of the lesion. With the decrease in the wound area, the ion balance and the electric field are gradually restored [20], indicating that the epithelial electric field plays an important role in the wound healing process. Electric fields are known to affect cell migration, protein synthesis, cell orientation, protein distribution, and activation [21–23]. In this study, we explored the application of an electric field to diabetic fibroblasts. Well-aligned spider silk (SS) and silkworm silk (SWS) fibers were prepared by electrospinning and served as a cell culture scaffold due to their biocompatibility and superior mechanical properties [24]. The electro-spun (E-spun) fibers mimicked the locally oriented ECM proteins in a native tissue to facilitate fibroblast polarization and activation [25, 26]. We have previously reported that incorporating a low dose of oxidized single-walled carbon nanotubes (SWCNTs) into fibrous proteins, such as collagen, not only reduce the toxicity of SWCNTs but also effectively modulate stem cell differentiation to neural cells [27–29]. A minute amount of CNT was also added to silk to produce electrically conductive, well-aligned, mechanically strong and biocompatible protein fibers [30]. Here, we delivered a low-voltage, square-wave pulse potential to fibroblasts grown on silk-CNT fibers to achieve renewed collagen and MMP synthesis favorable for connective tissue regeneration and chronic wound repair.

## 2. Materials and Methods

### 2.1 Materials

Major ampullate spidroin proteins 1 and 2 (MaSp 1 and MaSp 2) of dragline spider silk were obtained from Dr. Randolph V. Lewis of Utah State University. These proteins were purified from the milk of transgenic goats and mixed at a MaSp1/MaSp2 ratio of 4:1 to obtain optimized mechanical properties [31]. While most of the study was done using spider silk (SS) proteins, silkworm silk (SWS) and collagen (COL) were used for comparison and demonstrating their competence as alternative materials. Silkworm silk fibroin was extracted from cocoons of *Bombyx mori* silkworms (Aurora Silk, Portland, OR) and purified as per the earlier published protocol [32, 33]. COLI from calfskin was purchased from MP Biomedicals (Solon, OH). For electrospinning, the proteins were dissolved in 1,1,1,3,3,3-hexafluoro–2-propanol (HFIP) (Fisher Scientific, Pittsburgh, PA).

High purity single-walled CNTs were purchased from HELIX Material Solution (Richardson, TX), and were oxidized following the previously published protocol [27, 29]. A minute quantity of oxidized SWCNT was added to SS, SWS or collagen solution for electrospinning to generate protein-CNT composite fibers.

### 2.2 Electrospinning of Freestanding Protein Fibers

Aligned, freestanding silk and silk-CNT fibers were prepared using a home-built electrospinning system, as described in our previous work [24, 30, 34]. Briefly, a syringe pump (Harvard Bioscience Inc., Holliston, MA) was powered by a high-voltage power supply (Glassman High Voltage, High Bridge, NJ) to deliver a voltage of 25 kV at the tip of a blunt needle. The SS protein solution of 100 mg/mL was ejected at a flow rate of 1.2 mL/h for 30 s to produce continuous, high-density fibers that were collected and aligned across two parallel metal plates, placed 10 mm apart. For microscopic characterization and cell culture, fibers were transferred onto plastic substrates pre-cut from a Petri dish.

Our previous results [30] showed that E-spun fibers produced from 100 mg/mL SS with 0.05% CNT outperformed those with other CNT concentrations, thus they were used in cell stimulation experiments in this study. For the comparative study, 120 mg/mL SWS with 0.1% CNT and 100 mg/mL collagen with 0.5% CNT were chosen to prepare SWS-CNT and COL-CNT E-spun fibers with compatible properties and diameter similar to that of SS–0.05% CNT fibers.

### 2.3 Cell Culture

Passage 2 diabetic dermal fibroblasts (DDF) (of a type II diabetes patient) and non-diabetic dermal fibroblasts (NDF) (of a non-diabetic individual) were kindly provided by Dr. Eric Brey [35]. The frozen cells were thawed and cultured in Dulbecco’s Modified Eagle Medium (DMEM) supplemented with 10% fetal bovine serum (Corning, Manassas, VA), 1% MEM non-essential Amino Acid (Gibco, Carlsbad, CA), 1% MEM vitamin (Gibco, Carlsbad, CA) and 2% penicillin-streptomycin (200 U/mL) (Gibco, Carlsbad, CA) at 37°C in an atmosphere with 5% CO_2_. The medium was changed every other day and the cells were passaged every three days. The cells between 3^rd^ to 5^th^ passages were used and were seeded at a density of 8000 cells/cm^2^.

### 2.4 Electrical Stimulation of Fibroblasts

The fibroblasts were simulated using an in-house made portable electrical stimulation system [28]. The system was powered by a 9 V battery and the electrical potential was modulated by a NE555 square wave signal generator to deliver a voltage of 0.32 V at a frequency of 60 Hz. The potential was applied to cells via two gold wires, which were set 10 mm apart in the cell culture medium. Aligned freestanding fibers with pre-seeded cells were placed in the field for a desired period of time. To avoid protein aggregation, a 10-min interval was added for every 10-min stimulation to discharge the electrodes.

### 2.5 Cell Viability and Proliferation

Cell viability and proliferation tests were carried out by CellTiter 96^®^ Aqueous One Solution Cell Proliferation Assay (MTS) (Promega, Madison, WI) following the manufacturer’s instructions. Fibroblasts were cultured on various matrices in 96-well plates for five days. At days 0, 1, 2, 3 and 5, the cells were washed and then 100 μL fresh medium containing 20 μL MTS solution was added to each well. After 3 h incubation, the absorbance at 490 nm was recorded using a microplate reader (EL×808 Absorbance Reader, BioTek, VT) to examine cell proliferation. The MTS assay was also used to examine cell viability 24 h post-plating. The percentage of metabolically active cells on various matrices was determined with respect to the culture on blank Petri dishes.

### 2.6 Immunofluorescence Imaging

The cells grown on various matrices were fixed by methanol for 5 min at ‒10 °C followed by air-drying and then blocked with 10% bovine serum albumin (Sigma-Aldrich, St. Louis, MO) in PBS for 30 min. The cells were then incubated with primary antibodies for 60 min at room temperature in a shaker. Primary antibodies used in this study included rabbit anti-collagen I (Abcam, MA, 1:100), mouse anti-collagen III (Abcam, MA, 1:100) and mouse anti-α-SMA (Abcam, MA, 1:200 dilution). Secondary antibodies, purchased from Invitrogen (Carlsbad, CA), were used at 1:200 dilution and incubated with the samples for 1 h in a dark, humidified chamber. The slides were then subject to fluorescence imaging using a Nikon TE-U 2000 microscope. To rule out the influence of dye color in measuring the fluorescence intensity, Invitrogen™ Alexa Fluor™ 488 (Carlsbad, CA) and Invitrogen™ Alexa Fluor™ 594 (Carlsbad, CA) at 1:1000 dilution were used to calibrate the dye intensity. By switching the staining color for COLI and COLIII, we could also confirm the nearly identical binding affinity of the antibodies (< 5%). Quantitative analysis was carried out using ImageJ software. Background signals were subtracted to estimate the fluorescence intensity.

### 2.7 Total RNA Extraction and RT-qPCR

Total RNA was extracted from DDF and NDF cells using a PureLink® RNA Mini Kit (Ambion, Grand Island, NY). Reverse-transcription was carried out using a SuperScript® III First-Strand Synthesis System (Invitrogen, Carlsbad, CA). RT-qPCR was performed using an ABI Prism 700 with TaqMan® Gene Expression Master Mix and TaqMan® Gene Expression Assay (Applied Biosystems, Foster City, CA and pre-designed primers purchased from Life Technologies (Madison, WI). Primers against the following genes were used in this assay like that in our previous study [30]: *COL1A1* (Hs00164004_m1), *COL3A1* (Hs00164103_m1), *MMP2* (Hs01548727_m1) and *MMP9* (Hs00957562_m1). *GAPDH* (Hs99999905_m1) was used as an endogenous reference. Data analysis was carried out using the 2^−ΔΔCt^ method for relative quantification based on five replicate measurements. Student’s t-test was performed for statistical analysis.

## 3. Results

### 3.1 Protein and Gene Expression Profiles in Diabetic and Normal Fibroblasts

**Figure 1:**
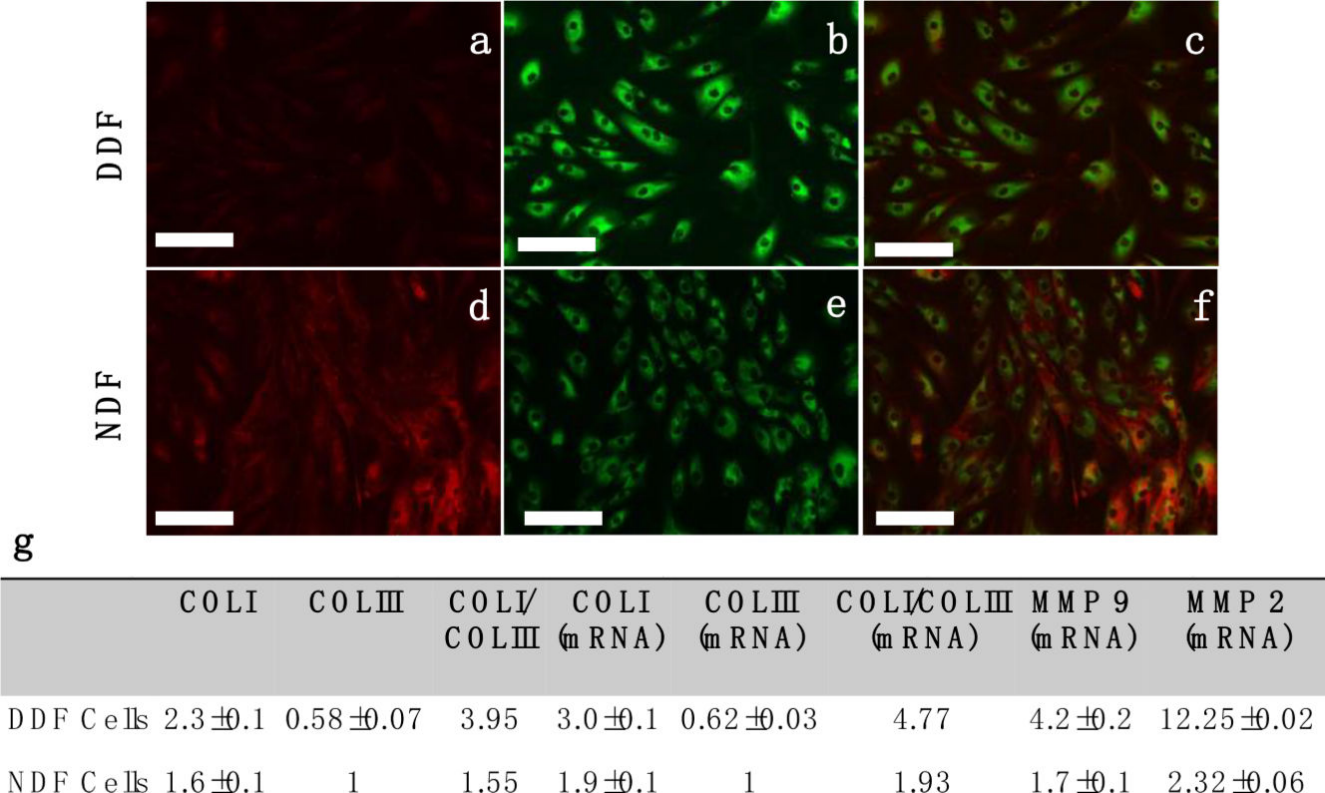
Immunofluorescence images of DDF (a,b,c) and NDF (d,e,f) cells staining against COLI (green) and COLIII (red). Images (c) and (f) show the co-staining of COLI and COLIII. Quantitative analysis of protein level COLI and COLIII expression (derived from immunofluorescence images) and gene level COLI, COLIII, MMP2 and MMP9 expression (derived from RT-qPCR analysis) are shown in (g). Protein and gene expression data were normalized with respect to COLIII expression in NDF cells. The cells were cultured on gelatin-coated petri dishes for three days before the immunofluorescence images were collected. Bar Size of the images: 150 μm

The difference between DDF and NDF cells in collagen synthesis was examined by COLI and COLIII immunostaining (Figure 1). Evidently, COLIII expression was lower in DDF cells than in NDF cells (0.58:1), alternately, COLI expression was higher in DDF cells than in NDF cells (1.48:1). Overall, the COLI/COLIII ratio was 3.95 in DDF cells and 1.55 in NDF cells. However, the total amount of collagen was similar in these two cell types. Consistent results were obtained on analyzing the expression level of genes (Figure 1g). Additionally, the expressions of MMP–2 and MMP–9, the key enzymes that degrade collagen in dermal tissues [36, 37], were found to be 5.3 and 2.5 times higher in DDF cells than in NDF cells. This is consistent with earlier reports of overly expressed MMP2 and MMP9 in diabetic patients [38–40].

We reported previously that hybrid fibers with less COLIII and excess COLI were stiffer and thicker [41, 42]. For wound healing, soft flexible COLIII is more desirable than COLI for the formation of granulation tissues to cover up the wound bed. The high COLI/COLIII ratio in DDF cells implies that these cells are incapable of supplying proper proteins to constitute granulation tissues. MMP–2 and MMP–9 overexpressions escalate the degradation of newly synthesized collagen molecules before they are properly assembled and deposited to form new tissues. This aberrant expression of collagen and MMPs is one of the key factors causing failure in wound healing in diabetic patients.

### 3.2 Matrix Mediated Cell Polarization and Activation

**Figure 2:**
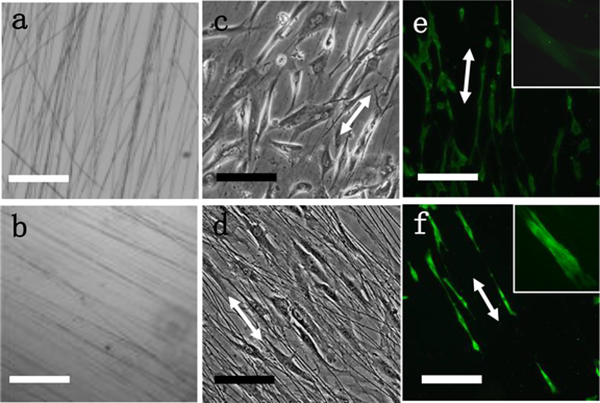
Optical images of E-spun SS fibers (a) and SS-CNT (0.05 w% CNT) fibers (b) as well as DDF cells polarized on these matrices at 12 h post-plating (c,d). The double arrows indicate the direction of aligned fibers. Immunofluorescence images illustrate α-SMA expression in cells cultured for 36 h on SS-CNT in the absence (e) and presence (f) of electrical stimulation. The insets highlight the stretching of actin filaments in individual cells. Bar size for a,b: 250 μm; for c,d: 50 μm; for e,f: 150 μm.

Attempt to correct the abnormal protein synthesis in DDF cells was made by modulating the matrix that supports cell growth. Unidirectionally aligned fibers provide physical cues to induce cell polarization and activation [24, 29, 36, 37]. In this work, SS and SS-CNT fibers were prepared by E-spinning. These fibers were unidirectionally aligned (Figures 2a, 2b), mimicking the local alignment of matrix proteins in native ECM. Consistent with our previous report [30], the addition of CNT to SS resulted in stiffer, thinner and better-aligned fibers. Nevertheless, an excess amount of CNT is known to cause cytotoxicity [38]. The cells on SS-CNT fibers proliferated at a similar rate as cells on pure SS fibers, with higher cell viability at lower CNT percentage [30]. SS fibers containing 0.05% CNT were chosen for the rest of the experiments due to their excellent biocompatibility and alignment.

The cells show polarization on E-spun fibers, in contrast to the random distribution of the spindle-shaped cells on gelatin (Figure 1). Further, the polarization was more significant on SS-CNT fibers than on SS fibers. We also collected the images of NDF cells and observed no significant difference in cell polarization between DDF and NDF cells. With the increase of culture time, the cells were seen to be more elongated on SS-CNT fibers than on SS fibers. The enhanced polarization of cells on SS-CNT is likely due to increased stiffness of the aligned matrix that promotes cytoskeleton stretching.

**Figure 3:**
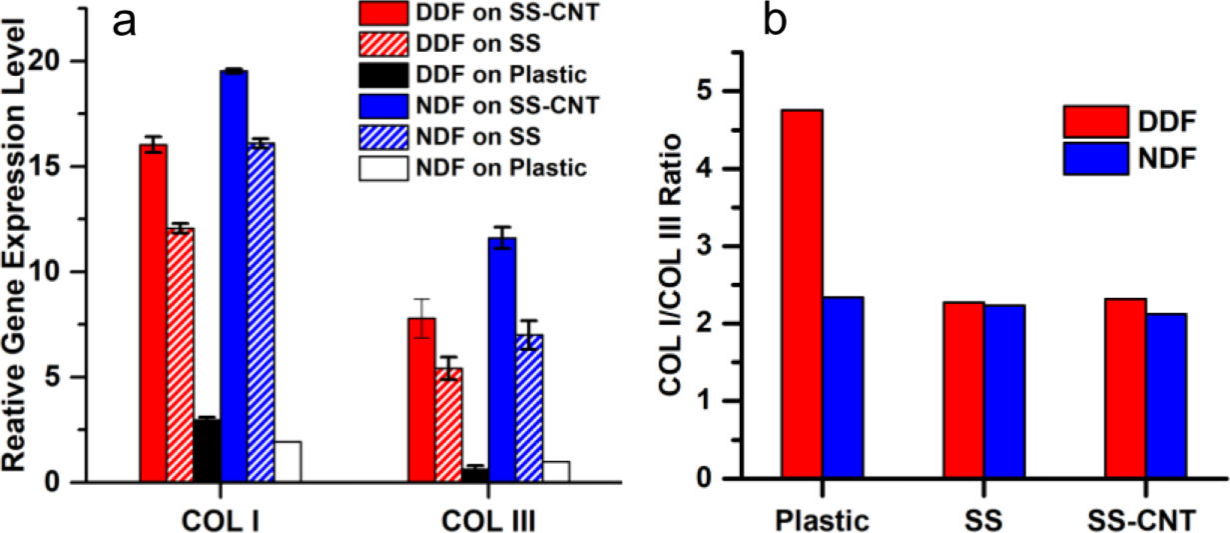
Comparison of COLI and COLIII gene expression profiles of NDF and DDF cells grown on plastic, SS fibers and SS-CNT fibers. a) Relative COLI and COLIII expression level of NDF and DDF cells after 5 days culture on various matrices. Log_2_-fold change was derived based on C_t_ calculation using GAPDH as a house-keeping gene and relative to COLIII expression in NDF cells cultured on plastic Petri dishes before reseeding on various matrices (set to 1). Error bars indicate standard error. b) Comparison of COLI/COLIII ratio in NDF and DDF cells after 5 days culture on the matrices. The ratio was derived from mean values in (a).

The RT-qPCR analysis was carried out for quantitative evaluation of the levels of COLI and COLIII synthesis by DDF and NDF cells, cultured for five days on SS-CNT fibers, SS fibers, and plastic Petri dish. Cells on SS and SS-CNT fibers synthesized a much higher amount of collagen than cells on plastic, and cells on SS-CNT fibers were more productive than those on SS fibers (p < 0.005) (Figure 3a). The augmentation was more significant for NDF cells than for DDF cells (p < 0.05). The ratio of COLI/COLIII was calculated and compared for the NDF and DDF cells cultured on various matrices and was found to vary over a narrow range (2.12 to 2.34) for NDF cells cultured on all three types of matrices (Figure 3b). In contrast, while the COLI/COLIII ratio was 4.76 for DDF cells on plastic Petri dishes, the ratio reduced to 2.27 and 2.32, respectively, for DDF cells cultured on SS and SS-CNT fibers. Therefore, aligned SS and SS-CNT fibers can not only boost collagen synthesis by fibroblasts but also renew the abnormal COLI/COLIII ratio in DDF cells, simultaneously retaining the normal COLI/COLIII ratio in NDF cells.

### 3.3 Cell Response to Electrical Stimulation

**Figure 4:**
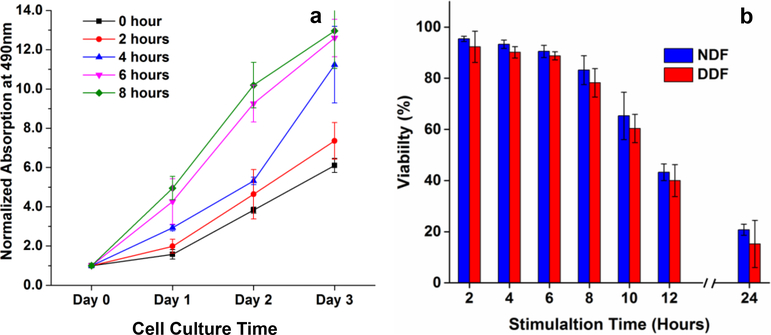
Effect of electrical stimulation on cell proliferation and viability. Cells confluent on plastic petri dishes were seeded on SS-CNT fibers for 12 h before the electrical stimulation was applied. a) Stimulation time dependence of DDF cell proliferation. b) Stimulation time dependence of cell viability for NDF and DDF cells at 24 h post-plating. The error bars indicate the standard error.

CNT increased the fiber conductivity, as evidenced by the decrease in fiber resistivity from 3.1×10^4^ Ω·m of pure SS to 6.4×10^2^ Ω·m of SS-CNT [30]. We explored the electrical stimulation of the fibroblasts by applying an electric field in the direction of fiber alignment. Stimulation time was optimized by examining cell proliferation and viability. As shown in Figure 4a, the rate of cell proliferation increased with the stimulation time. However, the increase in stimulation time from 6 h to 8 h did not lead to a further increase in proliferation rate. Although the data were generated using DDF cells, a similar proliferation profile was exhibited by NDF cells. To examine the cell viability, cells were stimulated for 2–24 h and subsequently cultured on the same matrix for 24 h. Cells without stimulation were used as control. The electrical stimulation for more than 6 h reduced cell viability to less than 90% (Figure 4b). Considering these factors, further experiments were carried out at 6 h electrical stimulation.

**Figure 5:**
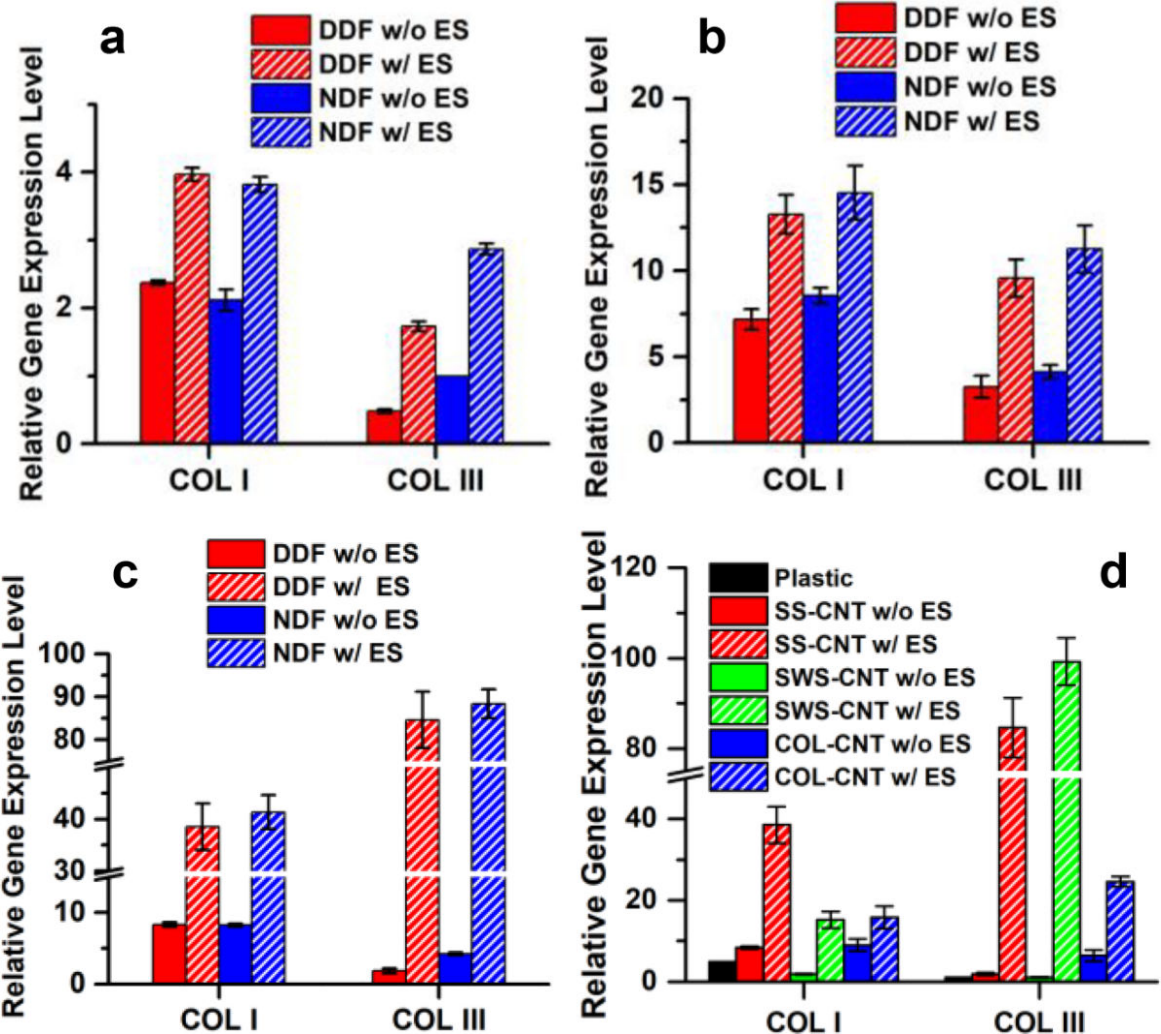
COLI and COLIII gene expression profiles of NDF and DDF cells grown on plastic (a), SS fibers (b) and SS-CNT fibers (c) for 12 h with or without 6 h electrical stimulation. Effect of DDF cell stimulation mediated by SS-CNT, SWS-CNT and COL-CNT is compared in (d). Log_2_-fold change was derived based on C_t_ calculation using GAPDH as a house-keeping gene and relative to COLIII expression in NDF cells cultured on plastic before stimulation (set to 1). The error bars indicate the standard error. ES: electrical stimulation.

RT-qPCR of COLI and COLIII expressions for cells grown on plastic Petri dishes, SS fibers and SS-CNT fibers in response to electrical stimulation reveal that the effect of electrical stimulation was three folds (Figures 5 a–c): (1) it boosted COLI and COLIII synthesis on all three matrices; (2) COLIII production was enhanced more significantly than COLI; (3) a dramatic enhancement was induced by SS-CNT fibers, leading to 5.2-fold and 42.7-fold increase in COLI and COLIII production by DDF cells, and 5.1-fold and 21.4-fold increase in COLI and COLIII production by NDF cells. Additionally, the electrical stimulation of cells on SS-CNT induced a decrease in COLI/COLIII ratio from 1.96 to 0.47 for NDF cells and from 4.49 to 0.46 for DDF cells. A low COLI/COLIII ratio is essential for the formation of granulation tissues, which is critical in wound repair.

Immunostaining of α-SMA, a marker protein of myofibroblasts, was carried out for cells grown on SS-CNT fibers (Figures 2 e,f). Electrical stimulation clearly induced a much higher level of α-SMA expression, and the actin filaments in the elongated cytoskeleton were uniform, parallel to each other, and orderly aligned. Thus, the fibroblasts were activated by electrical stimulation and transformed into myofibroblasts which are highly contractile and have increased collagen productivity.

The effectiveness of the matrix mediated cell stimulation was ratified by using alternative materials, silkworm silk, and collagen. E-spun SWS-CNT and COL-CNT fibers with similar properties and dimension as SS-CNT fibers were prepared (see Materials and Methods Section), and enhanced collagen synthesis due to electrical stimulation was observed for cells on all three matrices (Figure 5d). While the increase of collagen expression in cells on COL-CNT was not as significant as that on SS-CNT and SWS-CNT, the COLI/COLIII ratio in DDF cells was markedly reduced to 0.65. Similar to SS-CNT, SWS-CNT dramatically enhanced the collagen synthesis by fibroblasts and increased the COLIII production more effectively leading to a COLI/COLIII ratio of 0.17.

**Figure 6:**
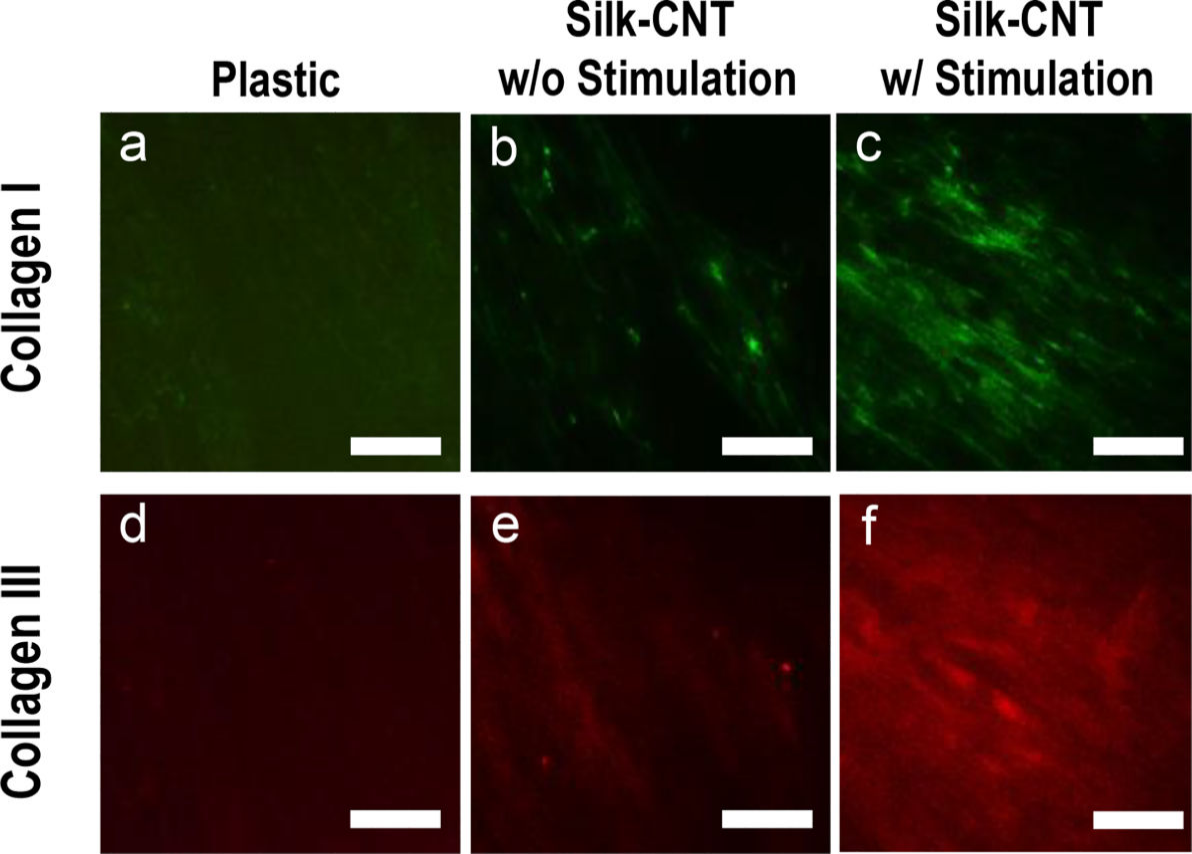
Effect of electrical stimulation on collagen synthesis and deposition by DDF cells on silk-CNT fibers. In the absence (b, e) and presence (c, f) of electrical stimulation, cells were cultured on silk-CNT fibers for 5 days before they were stained by COLI (a-c) and COLIII (d-f). Cells on the plastic substrate were used as a control. Bar Size of the images: 300 μm.

The deposition of collagen synthesized by the stimulated fibroblasts was examined by immunostaining of COLI and COLIII in five days old cells cultured on the matrices (Figure 6). The expression of both COLI and COLIII was remarkably higher in stimulated cells than in unstimulated cells. Quantitative analysis by ImageJ suggested that electrical stimulation induced a greater increase of COLIII expression than that of COLI, consistent with the results of gene expression analysis.

### 3.4 Post-Stimulation Cell Responses

**Figure 7:**
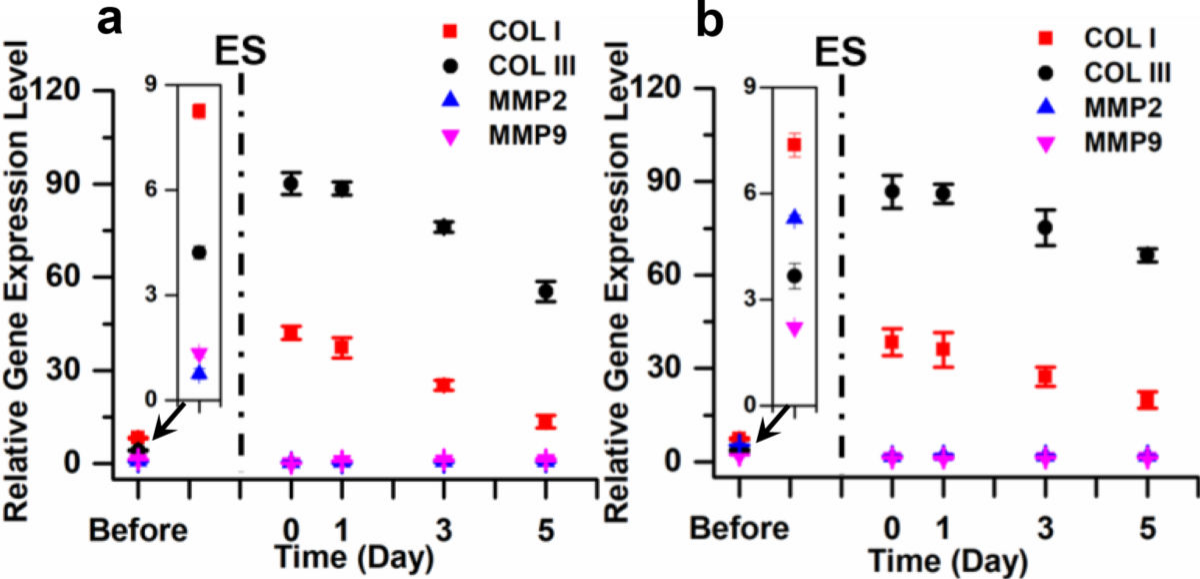
Time-dependent changes of collagen and MMP gene expression in NDF (a) and DDF (b) cells on SS-CNT fibers after 6 h electrical stimulation. After stimulation, the cells were transferred to plastic Petri dishes to perform RT-qPCR analysis at days 0, 1, 3 and 5 post-plating. The data at the time point “Before”, highlighted in the insets, represent data collected from the original cells before they were transferred to SS-CNT fibers. The error bars indicate standard error. ES: electrical stimulation.

It is critical that the renewed cells retain the function for a prolonged period after the termination of stimulation. This was studied by monitoring gene expression profiles in stimulated cells after they were transferred to plain plastic Petri dishes. RT-qPCR analyses of the cells at days 0, 1, 3 and 5 of culture were carried out to investigate the change in COLI, COLIII, MMP2 and MMP9 expression levels with time. Before stimulation, the expression levels of both MMP2 and MMP9 were higher in DDF cells than in NDF cells (insets of Figures 7a, 7b). Immediately after electrical stimulation, the levels of MMP2 and MMP9 dropped by 3.3 and 1.6 folds, respectively, in DDF cells, whereas no change was observed in NDF cells. From day 1 to 5, the MMP levels increased slightly in both cell types, although in the normal range as in NDF cells. Right after the stimulation, the levels of COLI and COLIII increased dramatically followed by a gradual decrease over time. Nevertheless, the levels of COLI and COLIII remained significantly higher than those before the stimulation. For instance, five days after the stimulation, the expression of COLI and COLIII was 2.7 times and 18.1 times higher in DDF cells and 1.6 times and 13.6 times higher in NDF cells. Importantly, the level of COLIII stayed higher than that of COLI. These results imply that the effect of stimulation can be preserved for an extended time period even after the cells are transferred to a completely different substrate.

## 4. Discussion

In this work, we discovered and achieved the transformation of DDF cells by aligned silk-CNT matrix-mediated electrical stimulation. In addition to synthesizing a significantly increased amount of COLI and COLIII, the transformed cells could overcome the low COLIII expression and high MMP expression. High MMPs limit the supply of COLIII required in large amounts for the formation of granulation tissues and causes a delay in overall wound healing process leading to chronic wounds [39]. Cells producing collagen at a lower-than-normal COLI/COLIII ratio is desirable, should these cells be transplanted for remodeling the wound’s stiff tissue to the normal level. The renewed protein expression profile of the stimulated cells signifies the novelty of this work and its potential application in treating chronic wounds of diabetic patients.

Incorporation of a minute amount of CNTs in silk fibers rendered the fibers stiffer and better aligned. It enhanced the cell polarization, consistent with our previous observations [24, 36]. Cell polarization induced cytoskeleton stretching and promoted the fibroblast-to-myofibroblast transition. Myofibroblasts contract using α-SMA-myosin complexes and, consequently, augment collagen production [40]. In response to the stiff silk-CNT substrate, the fibroblasts produce more COLIII than COLI to attain a softer matrix [29] and these effects were amplified by electrical stimulation. Electrical stimulation activates the TGF-β signaling pathway and triggers fibroblasts/myofibroblasts for increased collagen synthesis as well as reduced MMP production and activities that favor the release and deposition of fibrillar collagen [41, 42]. Silk-CNT fibers are 48 times more conductive than silk fibers and can more effectively transmit electrical signals to mediate fibroblast stimulation. Hence, the amplification was more dramatic in cells grown on aligned silk-CNT fibers than on silk fibers. This was verified by the high expression of α-SMA and the parallel aligned actin filaments in cells stimulated on SS-CNT (Figure 2f). During wound healing, the supply of a higher amount of collagen at a low COLI/COLIII ratio is desirable for granulation tissue formation. Impressively, electrical stimulation also suppressed the synthesis of MMPs by DDF cells (Figure 7a). This is critical as it warrants a proper balance between synthesis and degradation of collagen to allow the built-up of granulation tissues in the wound area. Further investigation is needed to delve into the mechanism of the distinctive MMP production by DDF and NDF cells in response to electrical stimulation. The effectiveness of the transformed DDF cells to accelerate wound healing was explored in a preliminary study using a subcutaneous mouse implant model, in which excisional wounds of 4 × 4 × 4 mm^3^ were treated by stimulated or un-stimulated cells embedded in silicone gel. The wound treated by stimulated DDF cells was observed to heal much faster than the controls of either the untreated wound or the wound treated by un-stimulated cells. The most dramatic effect was observed on day 1 of the treatment. Interestingly, no scar was formed in the presence of stimulated cells, likely due to the low COLI/COLIII ratio of the newly synthesized collagen. A systematic animal study to produce statistically analyzable results is expected to further elucidate the process and to modulate the stimulation process for improved outcomes. Particularly, the distribution and stability of the transformed cells, the quantity and quality of the newly formed tissues, the inflammatory response and neovascularization need to be investigated.

The research outcome can be potentially extended to develop the treatment of chronic wounds in diabetic patients. Instead of relying on cells from matching donors, dermal fibroblasts of a diabetic patient can be extracted and renewed *ex vivo*, then injected locally to the wound area of the same patient for treatments without the concern of immune rejection. We have shown that even though the transformed DDF cells were cultured on a plain plastic substrate for five days, they preserved their ability to produce collagen at a remarkably high level. Thus, it is conceivable that the transformed DDF cells can retain the functionality even after they are injected into a wound region and can impose a long-lasting effect on tissue remodeling to achieve accelerated wound healing. On the other hand, the locally injected cells would migrate and blend with the endogenous fibroblasts due to the high contractility and motility of myofibroblasts. The endogenous cells may synchronize with the transformed cells to acquire the myofibroblast phenotype and improved protein productivity. Conversely, the endogenous cells may “cancel” the functionality of the transformed cells via intercellular signaling. An alternative approach is to implant the transformed cells with the freestanding E-spun fibers. With the cell-imbedded SS-CNT fiber implant, patients can undergo physical therapy by locally applying a mild potential to re-stimulate the cells periodically when needed. Endogenous fibroblasts in the local region are also subject to ES and are expected to achieve improved functionality. Note that NDF cells can also be stimulated to produce a significantly higher amount of collagen at a low COLI/COLIII ratio. Thus, the same treatment strategies can be applied to the healing of wounds in non-diabetic patients.

Spider silk cannot be mass-produced and the process of producing synthetic spider silk protein is complex and expensive. The discovery that silkworm silk fibroin was equally effective to trigger the renewal of DDF cells is significant, as silkworm silk can be easily extracted and purified from raw silkworm cocoons which are abundant and inexpensive. Though COL-CNT is not as effective as SS-CNT and SWS-CNT, it inspires the exploration of other alternative materials, such as synthetic polymers, to generate CNT composite fibers or other forms of biocomposite fibers to achieve matrix-mediated stimulation of cells for tissue regeneration.

## 5. Conclusions

In this study, freestanding, unidirectionally aligned silk-CNT fibers, generated by the E-spinning technique, were employed to support fibroblast development. Their electrical conductivity enabled these fibers to effectively mediate the electrical stimulation of DDF cells not only to produce a high amount of collagen with low COLI/COLIII ratio but also to suppress the synthesis of MMPs and thus favor the formation of granulation tissue to accelerate wound healing.

The developed method can be applied to revive the function of fibroblasts in diabetic patients. Delivering these transformed autologous cells to the wounds of a diabetic patient is expected to renew the matrix protein expression profile, hence, recondition the microenvironment and support proper cell function, causing a long-lasting influence on effective wound healing. Fibroblasts are relatively abundant, easy to harvest, culture, expand and stimulate *in vitro*, making them an ideal and inexpensive source for cell-therapy of chronic wounds in diabetic patients and, in general, for connective tissue repair.
